# Data Evolution in Times of Crisis: an Organisational Mindfulness Perspective

**DOI:** 10.1007/s10796-022-10275-4

**Published:** 2022-04-29

**Authors:** Ger Flynn, Tadhg Nagle, Ciara Fitzgerald

**Affiliations:** 1 Health Service Executive and Cork University Business School, University College Cork, Cork, Ireland; 2 Cork University Business School, University College Cork, Cork, Ireland; 3 Cork University Business School, Cork, Ireland

**Keywords:** Organisational mindfulness, Critical events, Data, Resilience

## Abstract

The Covid-19 pandemic illustrates that we are never far away from situations that have a scale and impact, which are difficult to predict. Positioned at the intersection of crisis management and resilience, this insider case-study provides the opportunity for a more complete understanding of the organisation-adversity relationship (Williams et al., 2017), by focusing on the third Covid19 wave in Ireland (Dec 2020) and resulting response by an Intensive Care Unit crisis team. The study examines the evolution of seven data supply chains that were developed to support the ICU crisis team through the surge of cases which put the highest level of strain on the Irish health system since the pandemic began. The study focuses on 289 data reviews, which triggered 63 changes each requiring a new iteration of a data supply chain. Incorporating Organisational Mindfulness as the theoretical framework, the study provides an insight into the realities of data management during a crisis but also provides a rich awareness of the complexities of data management that often go unrecognised. In doing so, the study contributes the concept of ‘mindful data’, which aids managers to understand the key characteristics of resilient data supply chains. The study also provides a rare first-hand insight into how mindful data was constructed, presented, and evolved into an essential element within the critical care environment.

## Introduction

While examples of events that create existential organizational threats are numerous and varied (e.g. global economic downturns, natural catastrophes linked to climate change, and technology breaches) (Hällgren et al., 2018), the materialization of Covid-19 in 2020 as a pandemic illustrates that we are never too far away from situations that have a scale and impact that are hard to predict. Prior research in critical events and crisis management has leaned towards building resilience in organisations (Butler & Gray, 2006; Aanestad & Jensen, 2016; Su, 2017; Hällgren et al., 2018), providing better understanding of triggers and responses (Williams et al., 2017), and focusing on reflection and cognitive development as a response to these events (Lindh & Thorgren, 2016). Critical events, especially of a global nature, are traditionally an under researched area given the spontaneity of their occurrence. Thus we have a research problem of scant empirical studies on how actors assemble data elements, how this data is built during a crisis, and how this data is analysed within the decision-making process to manage the critical event. However, data collection and analysis during a critical event did feature in the German 2011 E Coli crisis. In particular, the study highlighted the length of for the data’s analysis (2 weeks) was a contributing factor in the delayed discovery of the source of the problem (Müller-Seitz & Macpherson, 2014). Furthermore, given the time sensitive nature of crises, King and Badham (2018) and Oeij et al. (2018) both called for a broad and comprehensive approach to Organisational Mindfulness and mindful infrastructure as a method of enhancing leader capabilities to operate swiftly and effectively in increasingly complex environments. This paper explores, through the constructs of Organisational Mindfulness, the evolution of data supply chains constructed during a crisis and how data was utilised in the management of the critical event.

Positioned at the intersection of crisis management and resilience, this insider case-study provides the opportunity for a more complete understanding of the organisation-adversity relationship (Williams et al., 2017). The case study focuses on a recent Covid19 surge event in Ireland (Dec 2020) and resulting response by the Acute Operations Critical Care Major Surge Group (ICU crisis team); a distinctive group assembled to ensure the critical care environment was adequately supported. Our study focuses on the research objective of exploring through the constructs of Organisational Mindfulness (OM) the evolution of data supply chains during a critical event to understand the realities of data management, resilience, and crisis management. The paper reviews the case study through the lens of Organisational Mindfulness to determine how mindful were the data elements selected and utilised when managing a critical environment during a critical event. The paper further explores if the evolution of these data elements allowed for swift effective actions within this environment. The outcome of this paper contributes to the literature of OM by extending the application of the constructs of Organisational Mindfulness as a possible effective lens to reliably guide the evolution of data during a critical adverse event.

Drawing on an ethnographic account of the lead author’s participation in a critical care special purpose group (ICU crisis team) during the Covid-19 adverse critical event, this paper explores the operational activity and data provided by this group. This assessment provides a unique insider account of how data elements specific to this critical care environment were chosen and how that data evolved during the event. The paper utilises the five constructs of Organisational Mindfulness to analyse how the data was chosen to manage the ICU environment. The paper further explores how the evolution of data supply chains can potentially improve reliability monitoring during an adverse critical event. The paper adds to the literature of critical event and crisis management as it provides a rare first-hand insight of how mindful data was constructed, presented, and how the evolution of data supply chains contributed to the management of essential elements within a critical care environment.

## Literature Review

### Organisational Mindfulness

Mindfulness at an organisational level refers to the capability of an organisation to develop a heightened awareness. Mindfulness can help people quickly discern threats and respond appropriately (Vogus & Sutcliffe, 2012; Weick & Sutcliffe, 2006). Mindfulness implies containing or minimizing the unexpected when it does happen, coping with problems as they occur, and containing not eliminating surprises (Aanestad & Jensen, 2016). According to King and Badham (2018) Organisational Mindfulness (OM) incorporates principles of anticipation (‘preoccupation with failure’, ‘reluctance to simplify’, and ‘sensitivity to operations’) and principles of containment (‘commitment to resilience’, ‘deference to expertise’). OM is accredited for developing awareness in volatile, uncertain, and complex circumstances such as healthcare delivery and its application is promoted as a method of achieving high reliability (Svalgaard, 2018; Bennett & Lemoine, 2014; Davidson & Begley, 2012; Weick et al., 2008). OM provides the ability to detect important aspects of the context and take timely, appropriate actions and increases an organisation’s ability to achieve reliable performance in dynamic environments. OM contributes to organisational learning by enabling the identification of errors and threats, creating multiple perspectives, providing contextualized interpretations and viewpoints, enabling attentiveness, situational awareness and tacit knowledge, stimulating the capability to analyse and learn from mistakes, and helping to deal with unexpected events (Becke, 2014).

In agreement, Oeij et al. (2018) suggested that mindful infrastructure is an antecedent of innovation reliance, as the motivation is to be continuously aware of unforeseen situations. This leads to stable cognitive processes for detecting possible errors, and to a variable pattern of activities for adapting to events that require revision. The benefits of individual mindfulness within an organisation is described as ‘a frame of mind in which an individual maintains a continuous attention to detail’. Collective OM provides benefits to an organisation through individualistic mindfulness and collaborative interdependencies through the collective mindfulness elements (Hales et al., 2012 p. 570; Matook & Kautz, 2008; VanVactor, 2012). Kirsh and Maglio’s (1994) classification of collective mindfulness highlights the presence of two types of operations in cognitive systems: epistemic operations (gathering information and interpreting it for subsequent decision making) and pragmatic operations (making decisions and acting on them). Such classification meshes well with environments of high reliability organising, wherein learning about a complex, dynamic environment and operating or acting on it take place simultaneously (Salivaara et al., 2019). According to Thatcher et al. (2018), a search of major academic journal databases revealed that IS research most often studies mindfulness at the organisational level, espousing the view that “greater mindfulness among decision makers changes the way in which mechanisms for environment scanning and information processing are used” (Fichman, 2004 p. 338). Mindful organising emphasises commitment to correct anomalies and potential problems by members with relevant expertise (i.e. commitment to resilience and deference to expertise), which together enable organisations to correct problems rapidly and capture opportunities for operational improvement (Vogus & Welbourne, 2003). However, OM is not without its challenges. Adopting a western pragmatic approach to both long-term and on-the-spot mindfulness interventions can be problematic if a clear understanding of the nature of mindfulness practices is lacking. Post a review of empirical studies on OM, Vu et al. (2018) propose that “right mindfulness” helps managers analyze, verify, and explore ethical dilemmas in organizations that require both an understanding of the current situation and the ability to learn from past experiences. For OM to be achieved, it must not be attached to any corporate purpose, deliberate strategy, or sophisticated hidden agenda (Vu et al., 2018).

Succinctly, there have been theoretical developments about mindfulness, specifically on the conceptualisation of the components (Weick et al., 2008) and the operationalisation of the measures of mindfulness (Mu & Butler, 2009). These constructs include: (i) deference to expertise, (ii) preoccupation with failure, (iii) reluctance to simplify interpretations, (iv) sensitivity to operations, and (v) commitment to resilience. According to Weick et al. (1999) and Weick and Sutcliffe (2006) the constructs can be understood as the following;

- **Preoccupation with failure**: awareness of failure in current operations and to mitigate this by building routines of anticipation from the occurrences of weak signals or small errors in critical areas.
- **Reluctance to simplify interpretations:** to restrict simplifications and complications in interpretations to get the right level of analysis for the situation being faced.
- **Sensitivity to operations:** the ability to capture the integrated big picture of operations at a higher level than the operational level, and comprising the collective mind beyond the individual operator.
- **Commitment to resilience:** the capacity to cope with unanticipated dangers after they have become noticeable. Resilience is the ability not only to recover from errors but also to manage with surprises in the moment, and to respond as they occur.
- **Deference to expertise:** refers to loosening the designation of who is the “important” decision maker to allow decision-making to migrate along with problems.

Recognised as an enabler to achieving a reliable organisation, mindfulness provides a wider response portfolio, better process awareness, and stronger accountability, which can lead to superior firm performance (Thompson, 1995; Weick et al., 2008). Given the typical adverse effects of service failures in healthcare delivery, healthcare organisations should strive to get it right first time (Hales et al., 2012; Ndubisi, 2012). Within this context, the ability to reliably identify and monitor critical data elements within an organisation providing early awareness of failures is of benefit. Subsequently the role that reliable data should play in an organisation ought to be paramount. Indeed, Dernbecher and Beck’s (2017) review on OM describes scant literature bringing together elements such as OM, high reliability, failure-prone contexts, and emphasises the role of data. The authors call for further research into interpreting OM as an accelerator, by which the influence of OM in the provision of data throughout a crisis can be explored. We answer this call by examining how the data supply chain evolves to manage the dynamic requests that emerge from the unexpected nature of a crisis.

### Data Evolution

The role of data management in crises as a solution as well as a cause is long known (Mitroff et al., 1987). For instance, the importance of timely reliable central data surveillance in the healthcare environment to assist in early detection of emerging large-scale problems was observed in an E-Coli outbreak. However, considerable delays in data supply from local health agencies reaching the federal state agencies delayed the realisation of the scale and scope of the crisis, limiting crisis management efforts (Müller-Seitz & Macpherson, 2014). More recently, research attention has shifted towards the use of big data in pre-empting and containing crises (Amaye et al., 2016; Watson et al., 2017; Akter & Wamba, 2019). Yet, we are still struggling to understand the realities and complexities of data (Fox & James, 2020; Jones, 2019) not to mind how best to utilise data innovations in crisis situations. For instance, the simple act of data visualisation was observed as an exercise of power for certain cohorts in a healthcare environment where they controlled data dissemination and decided what was monitored or reported (Huber & Gärtner, 2018).

To understand the realities, complexities, and evolution of producing data, researchers have used the data supply chain as a pivotal concept (Laney, 2018; Nagle & Sammon, 2017). Routed in early value chain research that explores the role of data in creating a competitive advantage (Porter & Millar, 1985; Rayport & Sviokla, 1995), Laney (2018) conceptualises the role of the information supply chain, which supports organisational processes in the creation and delivery of value. Furthermore, in developing the Data Value Map, a discursive template for building shared understanding around data initiatives, Nagle and Sammon (2017) explicitly call out four key stages in data production that runs through the centre of their model. The four stages incorporate the acquisition, integration, analysis, and delivery of data. The ideal process would entail the acquisition or capturing of relevant data on organisational entities, which is then integrated physically, logically, and conceptually. Analysis is then applied to the integrated data sets and delivered to data consumers who in turn make decisions and create organisational value. However, the realities of data are much different with common issues such as data silo’s (Enders et al., 2021), data/information overload (Li et al., 2021), and overall project failures (O’Neill, 2019). Nonetheless, the four stages of a data supply chain enables the visualisation and tracking of changes to data initiatives, which in this study is used to explore the evolution of crisis data.

## Research Methodology

**Fig. 1:**
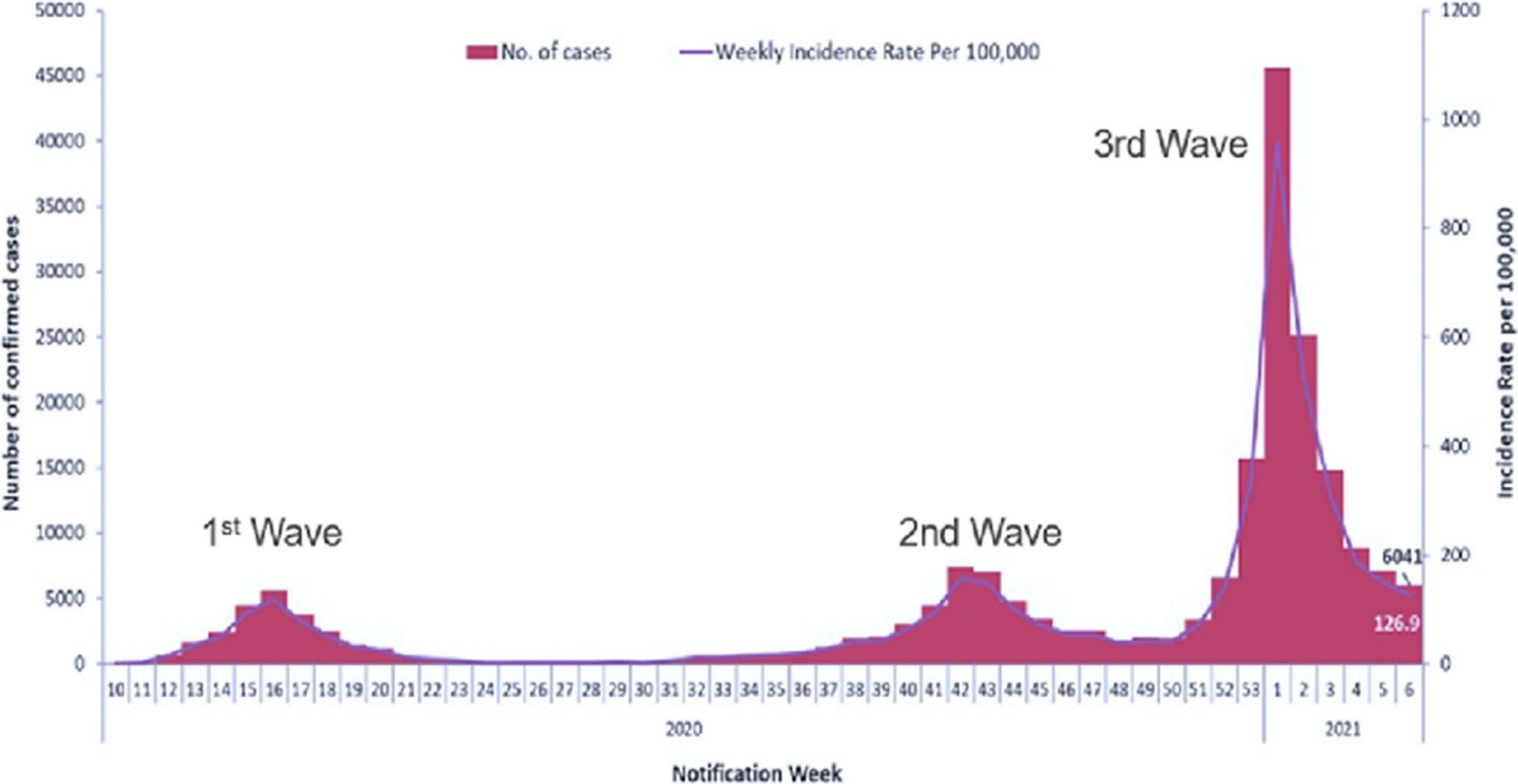
Rate of cases during the three Covid19 waves to hit Ireland, Lima, V., 2021

This study focuses on the 3rd Covid19 wave that hit Ireland in early 2021. As Covid19 cases started to rise, the Irish government put in place a national lockdown in late December 2020. However, with increased community infection, daily cases surged to over 8000 in January 8th 2021, the highest daily total since the start of the pandemic. As a result, the formation of an ICU crisis team was instigated by the HSE to oversee/coordinate a national critical care response in the context of an impending major hospitalisation and ICU admission surge. The period of operation of the ICU crisis team spanned the 11th of January 2021 to 2nd March 2021, during which time they met daily to monitor the impact of the pandemic. The crisis team was made up of multidisciplinary members, each possessing the specific skills needed manage the critical components of the national critical care network in Ireland. The network comprised of 26 individual Intensive Care Units (ICU), 2 Paediatric ICUs and 5 private hospital ICUs (Fig. 1).

The meetings focused on conducting a heuristic analysis, which required a data-driven and reliable picture of the situation on the ground, so that timely continuous dynamic succinct actions could be implemented to mitigate critical care environment failures over each 24-h interval. This is in line with the modus operandi of crisis teams to simultaneously learn about the complex dynamic environment and act on it, (Salivaara et al. 2019).

Furthermore, as outlined in the team’s Terms of Reference, the heuristic analysis focused on seven key areas: (i) Oxygen usage, (ii) ICU occupancy status, (iii) ICU staffing, (iv) ICU ventilator usage, (v) Non-Invasive Ventilator (NIV) and High Flow Nasal Oxygen (HFNO) usage, (vi), ICU Continuous Renal Replacement Therapy (CRRT) usage, and (vii) Patient Transfer Status. In essence, this study explores the evolution of the individual data supply chains required to provide a succinct presentation of the precise data necessary for the reliability of decisions taken in managing the crisis.

With the lead author a key member of the ICU crisis team and a senior manager at a national level for over 20 years in the Irish public health service (HSE), the case study was implemented from and insider inquiry perspective. Evered and Louis (1981) outline two approaches for conducting organisational research: (i) ‘inquiry from the outside’- researcher is an external actor from the focus of study, and (ii) ‘inquiry from the inside’- researcher is personally and actively involved in the focus of the research. Examining the two approaches, the authors found the knowledge gained through the later had increased validity and relevance to organisations. Indeed, while researching a crisis Müller-Seitz & Macpherson (2014) highlighted the benefit that inside inquiry could have been to their study by providing more ‘finer grained data’ (p. 606), albeit more difficult to plan with the unpredictability of crises. Through this insider inquiry approach, notes were taken on each of 36 daily meetings lasting one hour (on average), which took place over the total lifetime of the ICU crisis team. Moreover, these notes captured 289 data reviews that were conducted as part of the situational heuristic analysis but focused on the outputs of the seven data supply chains (see Table 1). Through these reviews it was possible to identify every data supply change requested and reason for requesting the change, which resulted in a new iteration of the supply chain (Appendix Fig. 9). This new iteration was actioned within the following 24-h reporting window and reviewed at the following daily meeting. Each of the changes were then analysed to provide a detailed insight into how each of the seven data supply chains evolved over the lifetime of the crisis team. Finally, these data supply changes were coded against the five components of OM to fulfil the research objective.

**Table 1:** Number of data reviews conducted during the lifetime of the ICU Crisis Team

Data supply chain focus	Oxygen usage	ICU occupancy	ICU staffing	ICU ventilator usage	NIV/HFNO usage	ICU CRRT usage	Patient transfer status
Decision supported	Advise pressure sites to prevent stress on pipework	Move patients to reduce pressure in ICUs	Reassign appropriate and sufficient staff to ICU	Reassign ventilators to pressure ICUs	Reassign ICU NIV’s to pressure sites	Reassign CRRT’s to pressure ICUs	Move patients to reduce pressure in ICUs and repatriate patients
Number of data reviews	48	54	36	30	33	54	34

## Findings

Analysing the 289 data reviews that took place during the tenure of the ICU crisis team, there were 63 change requests to the data supply chain, each resulting in a new iteration. Table 2 outlines the distribution of these changes across the seven data supply chains and the mindful influence of these changes. To further depict the mindful evolution of each of the data supply chains, seven vignettes are presented along with a visual representation of the supply chain. The vignettes depict specific examples of changes and the OM components they demonstrate.

**Table 2:** Organisational mindful analysis of each data supply chain iterations

		Data supply chain							
		Oxygen usage	ICU occupancy	ICU staffing	ICU ventilator usage	NIV/HFNO ventilator usage	ICU CRRT usage	Patient transfer status	Total iterations
Organisational Mindfulness	Sensitivity to operation	2	1	1	8	3	2	2	19
	Reluctance to simplify interpretations	7	1	2	2	6	0	0	18
	Preoccupation with failure	2	1	1	3	2	0	0	9
	Commitment to resilience	1	1	4	1	1	0	1	9
	Deference to expertise	1	1	3	1	1	0	1	8
	Total Iterations	13	5	11	15	13	2	4	63

### Oxygen Usage

**Fig. 2:**
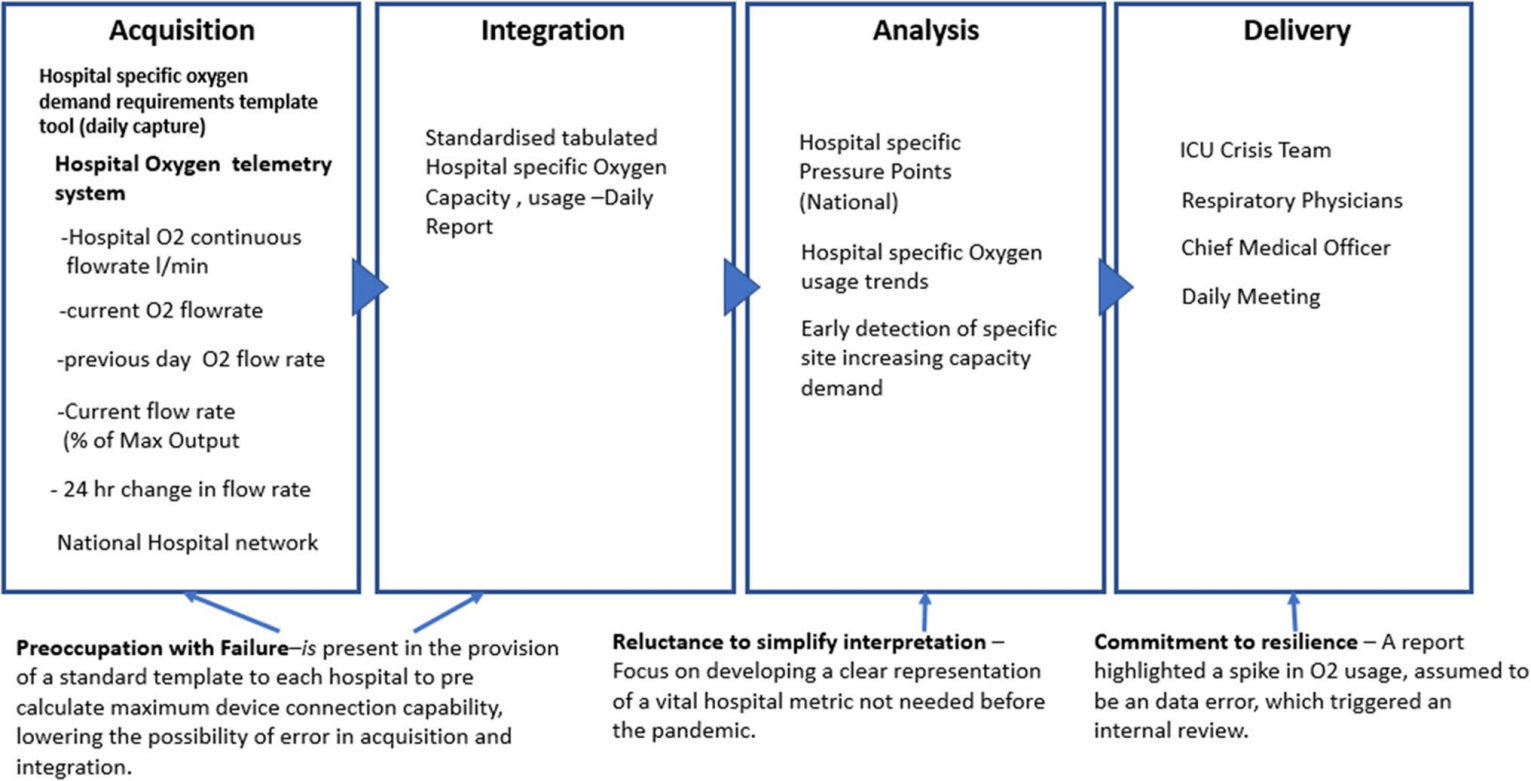
Visual presentation of the oxygen usage data supply chain vignette

The provision of oxygen is a vital ingredient in the care of patients with respiratory challenges. Extreme consumption of oxygen in hospitals resulting from the significant quantities of respiratory devices (simultaneously) in use, was a characteristic in the care for patients during the 1st surge in Covid 19 (March 2020). As demand in oxygen increases, the possibility of the flow of oxygen exceeding the capability of the hospitals oxygen pipework resulting in internal freezing within the pipe and blocking the flow of oxygen is likely, causing a catastrophic failure. Such scenarios occurred in hospitals located in Italy and the USA and as a result, hospitals became aware of the risk of excessive oxygen consumption. On the 6th of Jan 2021 a communication was issued to the HSE’s national estates office to review a spike in oxygen usage at an acute hospital. The spike was initially assumed to be a data error and triggered an immediate review of the data supply chain. This highlights embedded routines to handle errors when they occur and resulting *commitment to resilience*. Furthermore, the outcome of the review triggered an immediate requirement to develop a reliable hospital specific template tool to calculate the oxygen demand requirements dependant on the range and quantity of devices connected to the hospitals oxygen pipework system. Prompted by the ICU crisis team, 5 evolutions of the template ensued culminating in the completion of the template on the 16th of Jan 2021. The request to provide an accurate template tool to predetermine oxygen usage demands on each hospitals oxygen system, highlights a *preoccupation with failure* as it lowers the possibility of error in acquisition/integration that would occur if each site used idiosyncratic templates. Furthermore, the need to develop a metric that was never needed before the pandemic required a *reluctance to simplify* interpretations to ensure the right level of detail was provided through the category of devices, location, and their typical oxygen consumption rates (Fig. 2).

### ICU Occupancy

**Fig. 3:**
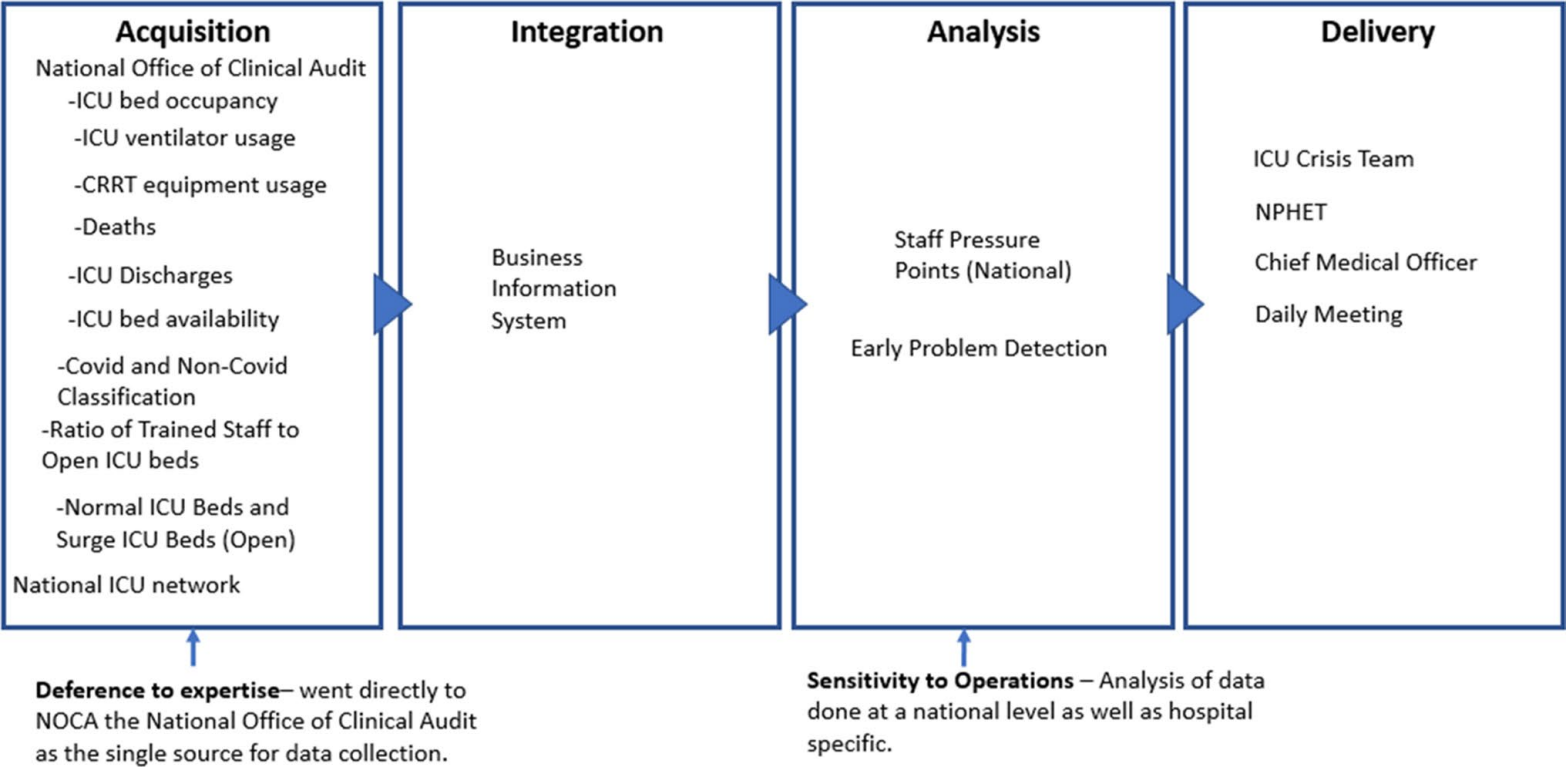
Visual presentation of the ICU occupancy data supply chain vignette

ICU occupancy and ICU ventilator usage data was primarily provided through the specialised audited ICU reporting system in the National Office of Clinical Audit (NOCA). The ICU crisis team seeking reliable data directly from trusted providers of national data highlights the mindful construct *deference to expertise.* Daily ICU activity monitoring by the ICU crisis team across the national network via the specialised audited ICU reporting system provided reliable data for assessing activity. Furthermore, that need to analyse this data at a national context depicts a *sensitivity to operations.* This data source increased the ability to gain a national context and the interconnectivity between each of the ICU sites, enabling operational interventional decisions to be triggered, such as the movement of patients. During the period in which the ICU crisis team operated (11th January 2021 to 2nd March 2021), the normal maximum ICU patient occupancy rose from a 289 to an average daily surge occupancy of 331. To alleviate pressures in specific ICUs during this period, 108 patient transfers occurred in total from occupancy pressurised ICUs (Fig. 3).

### ICU Staffing

**Fig. 4:**
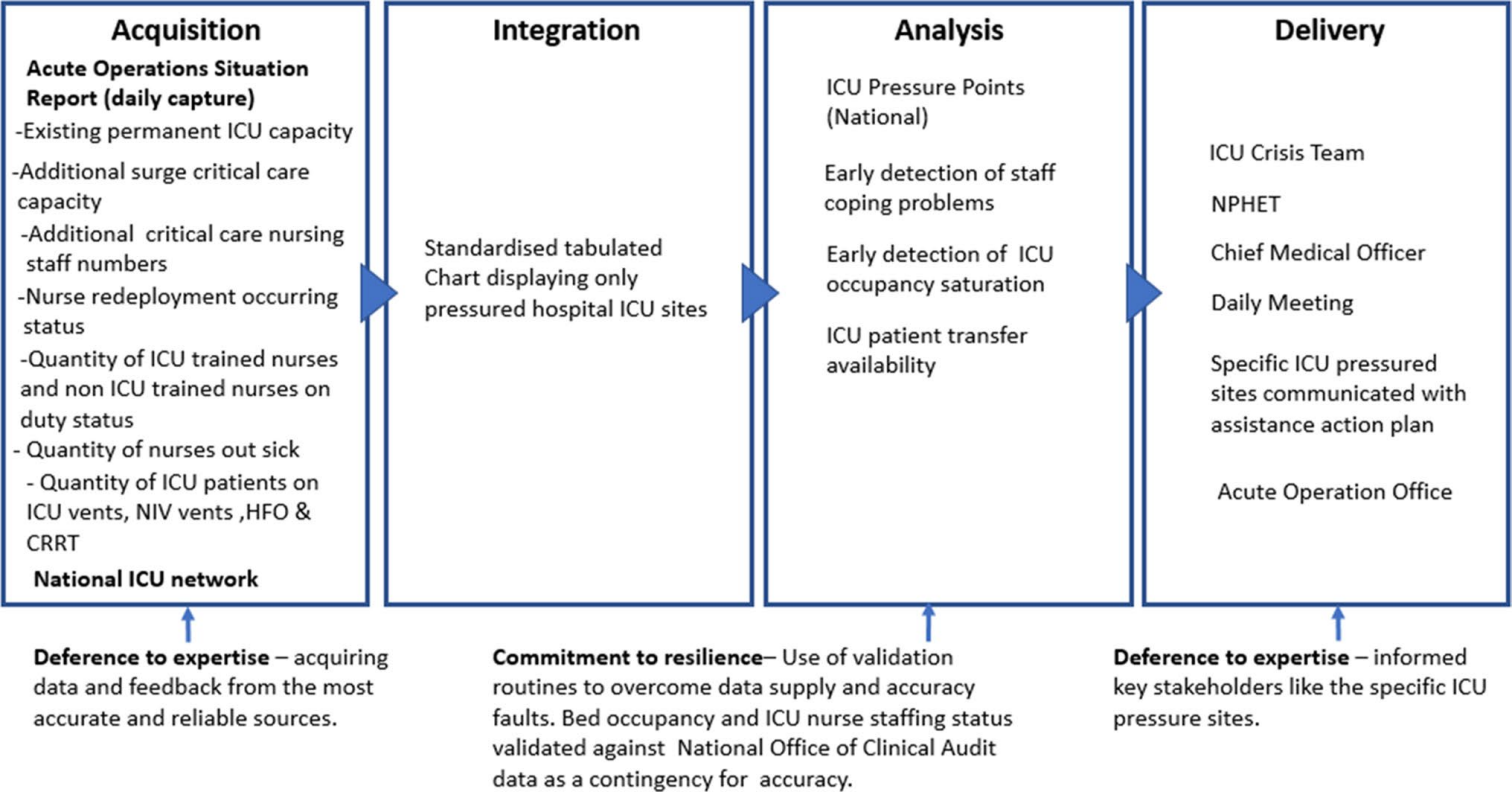
Visual presentation of the ICU staffing data supply chain vignette

Daily communication by the ICU crisis team with each ICU site across the national ICU network during the period 11th January 2021 to 2nd March 2021, provided a robust informative data source on the ICU staffing status. This data contained details such as staff skill mix, staff out sick situation, and staff coping status to the ICU crisis team. The feedback from these ICU communications provided a robust informative data source for reporting the current range of pressures experienced by the ICU clinical staff and articulated the specific assistance they sought. *Deference to expertise* by the ICU crisis team is revealed through two stages (1) seeking feedback directly each day from the clinical staff at each the ICU sites and (2) presenting reliable situational data to the key stakeholders. Continual feedback during the period 13th January to the 16th of January resulted in 3 new iterations of the data supply chain for the ICU crisis team. These data evolutions improved the ability of the ICU crisis team to capture specific data such as additional ICU bed staffing capacity/capability, elective surgery activity and appropriate staff competency ratios. During the period 12th January to the 22nd of February 120 separate requests for assistance were received by the ICU crisis team from various ICUs. With a data-driven insight into the situation the chair of the ICU crisis team contacted the hospitals most at risk and requested adequate nursing resources be made available to the ICU through the suspension of elective surgery and other elective services. Identifying such problems before failures occur and alleviating deficiencies can contribute to bolstering resilience in an organisation. *Commitment to resilience* was also demonstrated when the accuracy of the bed occupancy and staffing data was compared to the NOCA reporting system data for any data variations that existed. This data validation routine reconciled any inaccuracies that would have otherwise led to supply failures and poor decisions (Fig. 4).

### ICU Ventilator Usage

**Fig. 5:**
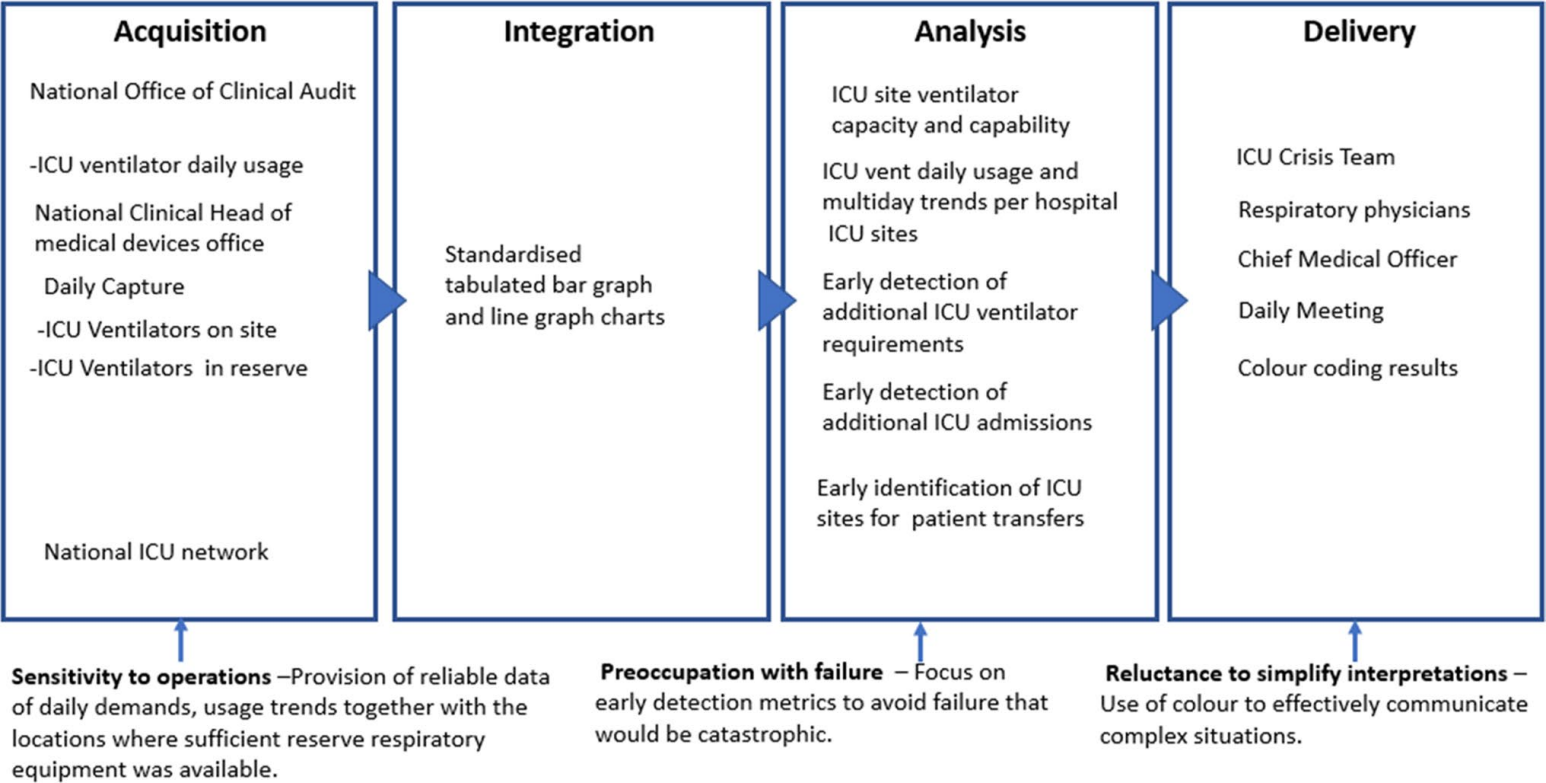
Visual presentation of the ICU ventilator usage data supply chain vignette

Data on ICU ventilator capacity per ICU was provided at the inauguration of the ICU crisis team on the 11th of January 2021. This data presentation necessitated further refinement as a measurement of daily ICU ventilator usage and reserve capacity per ICU. As the assurance in data reliability was paramount, the specialised audited ICU reporting system containing ICU ventilator usage was referenced, with the first data presentation containing ICU ventilator usage and quantity in reserve occurring on the 19th of January 2021. The resulting data requests suggest a *sensitivity to operations* by the ICU crisis team, as the heuristic analysis required eight evolutions of the data supply chain to provide a precise and interconnected presentation of the daily demands, which included usage trends with the locations where sufficient reserve respiratory equipment was available across the entire ICU network. In addition, the HFNO and NIV trend data was added to the ICU ventilator trend data to highlight the sites where ward located respiratory equipment usage was increasing, which could result in possible increases in ICU admissions at that site. The combined presentation of this data allowed decisions to transfer ICU patients to other ICUs and generate capacity when HFNO and NIV usage trends data were increasing at a specific hospital site. The evolution of data occurred during the period 19th January to the 2nd of March 2021 and began with colour coding to improve presentation clarity and help communicate the complexity of the situation in an effective manner (*reluctance to simplify interpretations*). For instance, ICU ventilator usage was coded in yellow and in reserve in green per site, which evolved into multi day trends displaying the highest daily ICU ventilator usage sites coupled with multiday day trend usage of HFNO and NIV. Furthermore, as the 3rd Covid19 wave progressed the focus of early detection metrics of the daily demand of respiratory equipment usage became a key focus; *a preoccupation with failure* by the ICU crisis team so that respiratory demand was met with availability to avoid a catastrophic failure (Fig. 5).

### NIV /HFNO Usage

On the 14th of January 2021 the ICU crisis team requested data on High Flow Nasal Oxygen (HFNO), Non-Invasive Ventilator (NIV) availability, and usage outside ICU so that patient respiratory treatment capacity could be understood. The external ICU focus of this data requirement indicates a *sensitivity to operations* as the data, which was integrated in a spreadsheet provided a reliable insight to the ICU crisis team. Continuous heuristic reviews of the returned data by the ICU crisis team resulted in six data supply chain changes during the period 19th January to 3rd February*. *These resulting iterations indicates a *reluctance to simplify interpretations* to deliver data with the appropriate focus and granularity, such as the current daily usage per site, location of reserve HFNO /NIV equipment, and multi day trends in usage.

**Fig. 6:**
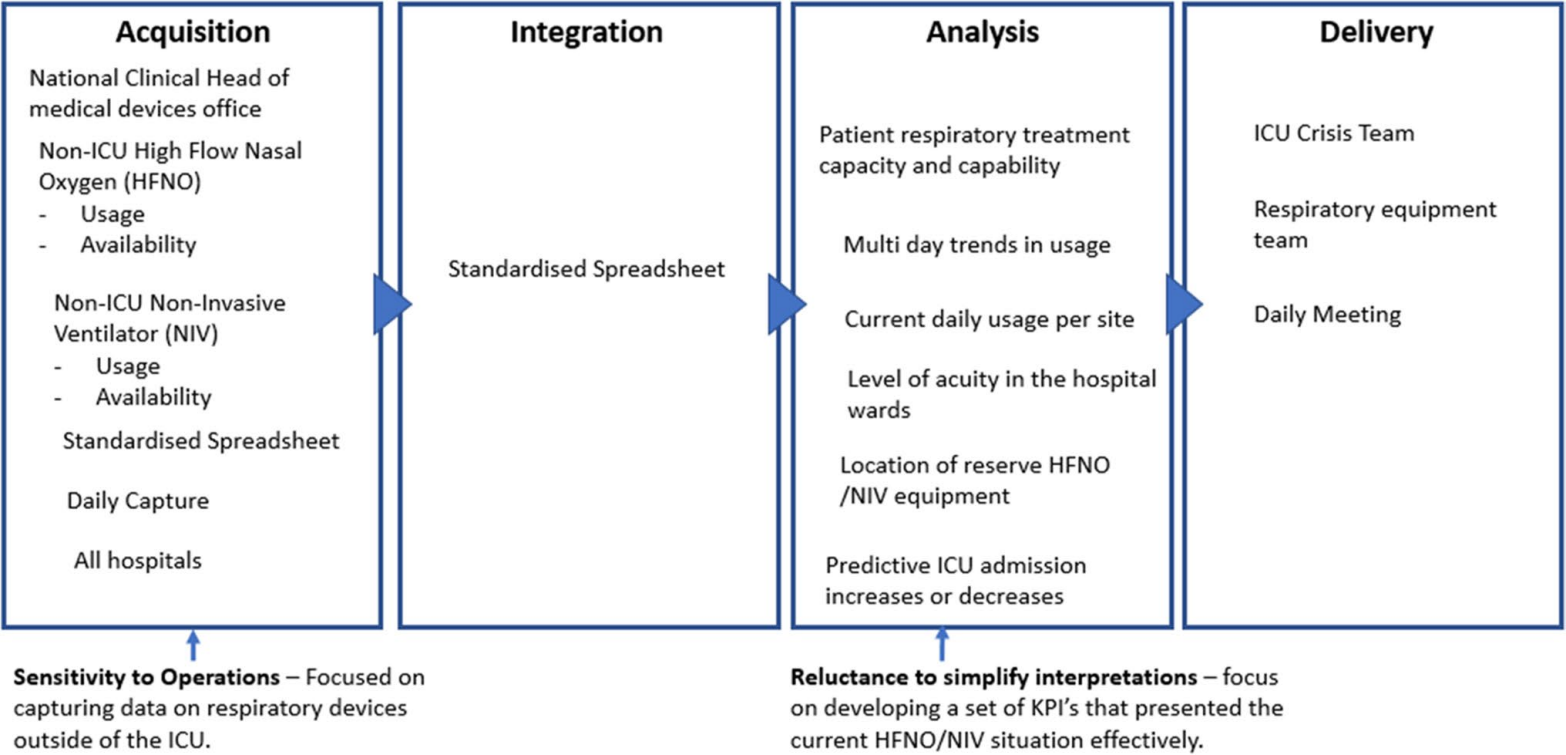
Visual presentation of the NIV/HFNO usage data supply chain vignette

The data supply chains provided valuable KPI’s that enabled the crisis team match demand for 117 HFNO /NIV devices against ICU sites that had reserve equipment during the period 16th January to the 12th of February 2021. The data also provided the respiratory clinicians a national overview of HFNO /NIV equipment usage outside of the ICU for the very first time (Fig. 6).

### ICU CRRT Usage

**Fig. 7:**
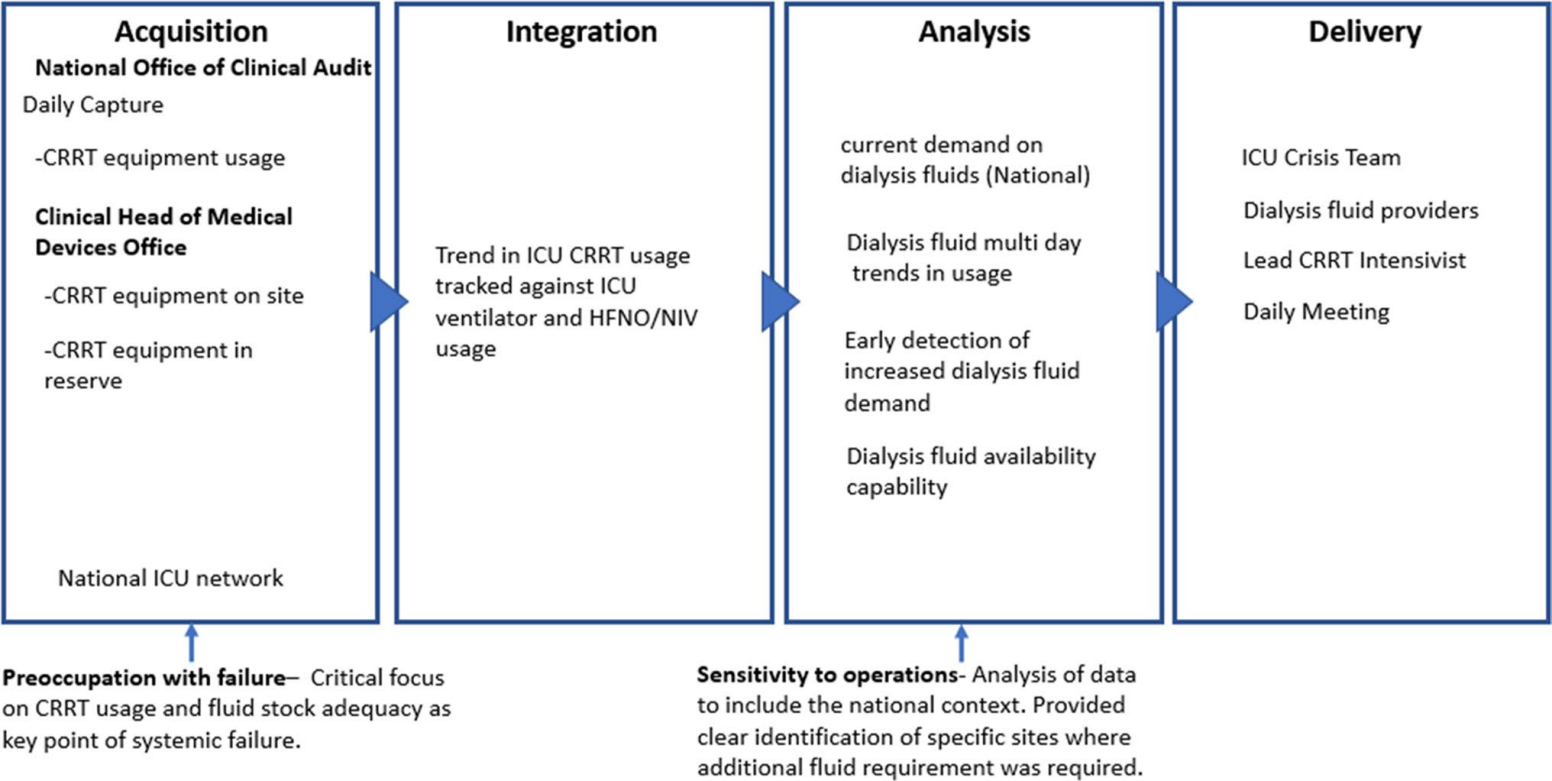
Visual presentation of the CRRT usage data supply chain vignette

The ICU crisis team received a communication from a HSE dialysis fluid supplier on the 20th of January 2021 of potential shortages in dialysis fluids because of high demand for fluids across Europe. Continuous Renal Replacement Therapy (CRRT) requires specific dialysis fluids to provide renal support for critically ill patients in an ICU. Because of possible disruption in dialysis fluid supply, the ICU crisis team sought data on the daily usage of CRRT machines to understand the current demand on dialysis fluids. As assurance in data reliability was paramount, the specialised audited ICU reporting system containing ICU CRRT usage was referenced with the first presentation of this data element to the ICU crisis team occurring on the 21st of January 2021. This action suggests a *preoccupation with failure* by the ICU crisis team as the feedback provided a reliable indication of current demand which was compared with dialysis fluid availability to mitigate against a systemic stock inadequacy failure. *Sensitivity to operations* by the ICU crisis team is evident on the 24th of January 2021 when the team sought an evolution in the CRRT data provided. This data evolution provided a national overview of CRRT usage trend data so that an increase or decrease in demand for dialysis fluids could be tracked as the 3rd wave surged through the country (Fig. 7).

### Patient Transfer Status

**Fig. 8:**
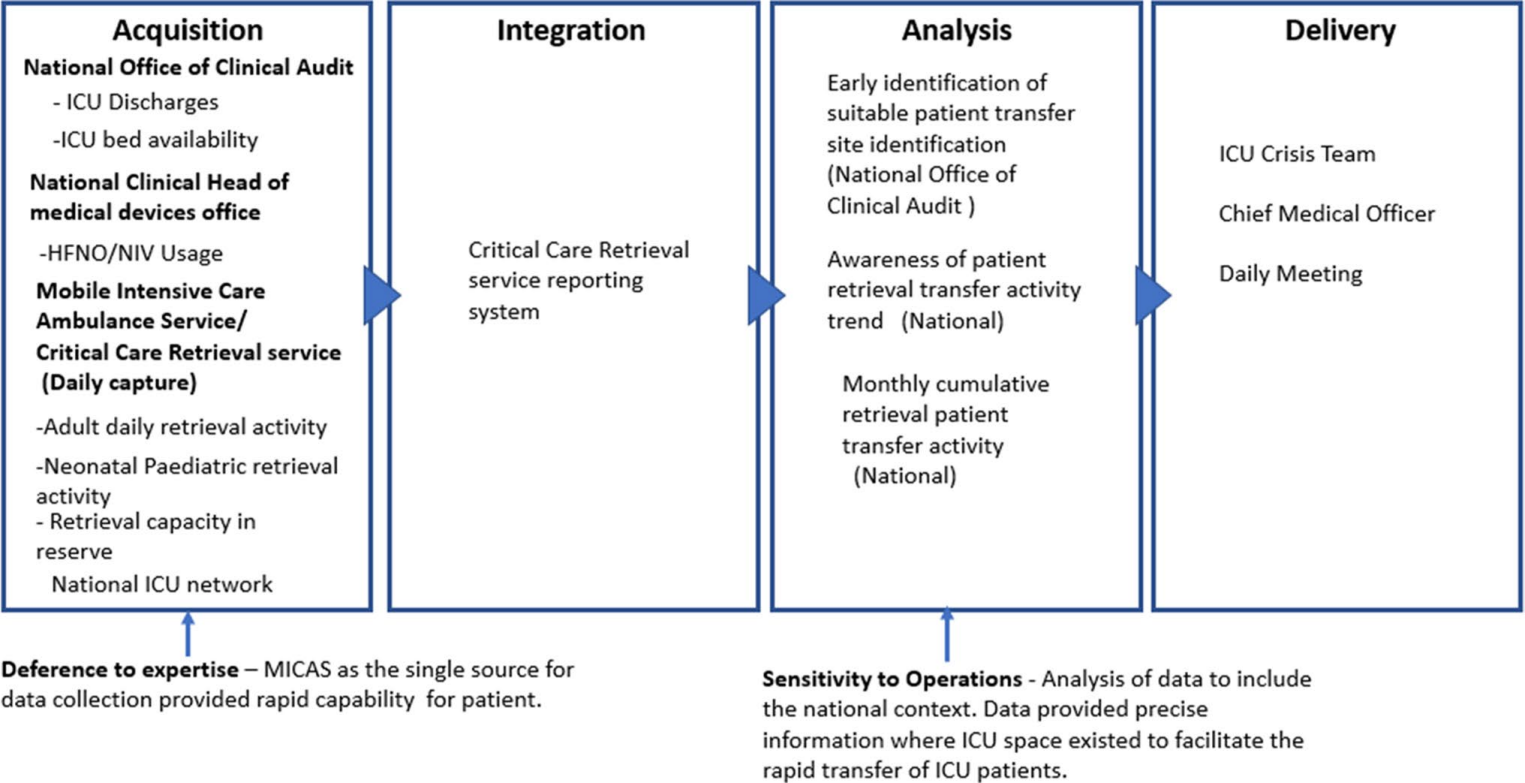
Visual presentation of the patient transfer data supply chain vignette

Daily comparison of the ICU staffing status with the specialised audited ICU reporting system provided reliable data on which sites were experiencing current occupancy pressures. Review of these data elements in conjunction with the HFNO/NIV trend data provided a reliable early warning tool to the ICU crisis team to predict ICU occupancy requirements for specific ICUs. During late January 2021 a cluster of significant infection rates in a specific regional area resulted in an episodic increased rate of ICU admissions to several acute hospitals in that region. The ICU crisis team’s access to the audited ICU reporting system allowed for reliable instant data where ICU capacity existed within the national ICU network. As in the other data supply chains, data was sourced from those with accurate insight into the situation (*deference to expertise*), which in this case was the ambulatory ICU patient transfer teams (MICAS). The interconnected nature of patient transfer required the integration and analysis of other operational data sets across ICU’s (e.g. ICU occupancy, HFNO/NIV usage) to enable rapid decisions on patient transfers to support pressurised ICU sites. As a result of this *sensitivity to operations*, the data supply chain supported a total of 49 ICU patient transfers that were completed during the period 26th January to the 9th February 2021. The ICU crisis teams direct access to specialised ambulatory ICU patient transfer teams, allowed for data-driven decisions to be completed within a short period of time with the outcome of these actions reported within a 24 hour period (Fig. 8).

### Summary

Ex﻿amining the number of iterations, sensitivity to operations and reluctance to simplify interpretations demonstrate the strongest influence. This would broadly align with the core functions of a data supply chain, which is to capture a comprehensive picture of operations and deliver consumable insights. However, the frequency of changes across the five categories, it is possible to understand locus of attention for developing resilient data supply chains in comparison to other crisis management activities. Throughout the study there is evidence of a significant effort to pre-empt and anticipate data failures, which focused on the early stages of the supply chain (see Table 3). This ensured the right data was captured, from the right people, and in an accurate fashion, which reduced the risk of errors or failures in its provision and use. If the data were poorly framed then even good execution of the decisions taken based on the data would typical not lead to good results (Karelaia & Reb, 2015).

**Table 3:** Organisationally mindful data characteristics

Mindful constructs	Mindful data characteristics	Dominant data supply chain phase	No. of iterations
Sensitivity to operations	Ensuring data completeness and analysis that incorporates the interconnected nature of crises (e.g., all sites, all devices).	Acquisition and analysis	19
Reluctance to simplify interpretations	Providing the right balance between accuracy, clarity, and simplicity of the results to ensure the effective consumption of the data (e.g., multiple iterations of analysis and delivery phases).	Analysis and Delivery	18
Preoccupation with failure	Pre-empting any data errors at source that may arise from gaps in the data or ad-hoc acquisition strategies. (e.g., providing and requesting sites to complete standard templates to enhance accuracy of data).	Acquisition, Integration, and Analysis	9
Commitment to resilience	Embedding of routines that are triggered if an error is flagged. (e.g., validating data across other sources)	Analysis and Delivery	9
Deference to expertise	Ensuring data is captured and supplied to the key stakeholders (e.g., the data creators and data consumers).	Acquisition and Delivery	8

## Discussion

### Organisationally Mindful Data

During the ICU crisis response, there were many organisationally mindful behaviours that focused on the operational aspects of the ICU network. For instance, there was a massive effort in procuring extra ventilators and PPE to increase the supply of healthcare services. However, this study focuses specifically on the supply of data and the mobilised routines that ensured the data component of the crisis response did not fail. The data focus chosen per ICU site across the entire national ICU network provided the ICU crisis team with a reliable overview of key aspects such as staff availability, ICU bed occupancy, equipment, and oxygen consumption. Continuous monitoring and granular evolution of the data was essential for reliable and effective decision-making that mitigated identified emerging risks. In essence, this study provides a description of mindful data by detailing the inductive mindful process through which the data was produced. Moreover, incorporating the data supply chain (Laney, 2018; Nagle & Sammon, 2017) as unit of analysis has provided insights at the intersection of crises (Williams et al., 2017), resilience (Weick & Sutcliffe, 2015), and data management/governance (Khatri & Brown, 2010; DalleMule & Davenport, 2017). Furthermore, the inside inquiry approach provided an opportunity to assess the realities of data management that would be otherwise difficult to attain. For instance, the relentless pace and rigour invested into each data supply chain clearly depicts the separation between data in principle from data in practice (Jones, 2019). As such, it provides a glimpse of the “gulf between the way data are commonly presented and the way that they are produced and used in practice”. Indeed, the study provides a “richer awareness of the complexities of data and the often unrecognized work that is involved” (Jones, 2019 p.15). For example, examining the locus of data supply changes from a data management perspective, the study depicts a data strategy that is more defensive in nature. A defensive data strategy is one that focuses on tasks such as accuracy, standardisation, and a single version of the truth (DalleMule & Davenport, 2017). Even though hospital data strategies are naturally defensive, the dynamic nature in which the data was captured, required an added defensive focus to ensure the data was fit-for-purpose within a crisis scenario. There were offensive characteristics visible in the iterations (e.g. implementing predictive analytics), yet they did not extend to advanced analytical techniques. The lack of emphasis on these advanced analytical techniques would suggest a limited need for big data technologies. Furthermore, given the critical nature of the study, the value in conducting the analysis was not symbolic as is the case for many advanced data applications (Grover et al., 2018). As a result, the study provides some insights into the need for big data in a crisis situation but such insights are tempered by the scale of the 3rd Covid19 wave, which is not at the scale of a natural disaster, such as an earthquake. Nonetheless, it gives an insight into the types of contingencies put in place, the level of resources needed, and process of developing data agility, all of which are research gaps within the domain of big data and disaster management (Akter & Wamba, 2019).

According to King and Badham (2018) the principles of anticipation relate to the OM concepts of preoccupation with failure, reluctance to simplify, and sensitivity to operations, while the principles of containment are associated with commitment to resilience and deference to expertise. However, containment was only evident in commitment to resilience in this study when the crisis team triggered validation and data check routines after errors were flagged. The evidence of deference to expertise was mainly from an anticipatory perspective, which sought to engage the data creators and consumers at either end of the data supply chain. This would be in line with the notion that the two critical points in the lifetime of data are when it is created and when it is used (Redman, 2008). It also highlights the importance of people at these time points, regardless of their rank. In particular, the frontline staff were contacted on a daily basis not only as a source for data but also as an opportunity to empathise. Indeed, recognising the role of emotion in crisis events is something that should not be overlooked (Williams et al., 2017), especially in data processes that may be seen as technical in their orientation and not human at the core.

The term ‘mindful data’ implies an awareness that being resilient requires resilient and reliable data processes. Just as poor data gives you poor decisions, unreliable data processes limits resilience efforts in an organisation. Through the iterative nature of the data supply chain development, it is possible to see the influence of mindful routines in building the resilience and reliability of the process. As such, ‘mindful data’ goes beyond Khatri and Brown (2010) framework for data governance design and provides a more business and situation specific perspective as to how to best manage data for successful outcomes. It also differentiates between data governance routines that lean heavy on the ostensive role of routines through lengthy policy documents versus the performative nature of mindful behaviours that give a clear picture of the level of data management enacted (Feldman & Pentland, 2003). For instance, the study demonstrates how mindfulness can mitigate the limitations of evidence-based management in terms of who determines what data to acquire as evidence, or what counts as a better decision (Hornung, 2012), or the objectivity and referentiality of the data utilised (Jones, 2019). As presented in the case, the fluid nature of what was important shifted continuously, which required immediate feedback loops overriding any individual or groups claim on determining priority. In fact, the data supply chains that emerged provided new operational insights, such as a national overview of HFNO /NIV equipment usage outside of the ICU, which was provided to respiratory clinicians for the very first time. This data source was valuable in determining the level of acuity in hospital wards and depending on the trajectory trend provided an early indication of impending ICU admission increases or decreases.

## Concluding Comments

This paper makes a number of theoretical and practical contributions. Firstly, this paper contributes the concept of ‘mindful data’, which denotes the output of applying mindful processes/routines to data analysis. Moreover, incorporating the data supply chain has provided insights at the intersection of data management/governance, crises and resilience. Secondly. this study reveals synergies between the components of action learning (group focusing on a problem with the power to solve it) mindfulness (provides reliability in the choices made) and reflection in action as the group endeavours to manage multiple complex environments as one (Kremser & Blagoev, 2020). OM components such as sensitivity to operations allowed for improved understanding of the interdependences and interrelations of many aspects of an organisation (Weick & Sutcliffe, 2015). Reluctance to simplify interpretation provided the ability to holistically evaluate the critical environment with an emphasis on collecting more granular information and broadening the search for alternative explanations for what may be ignored or discarded, thus providing the ability to uncover the emergence of potential problems in their infancy (Su, 2017; Weick & Sutcliffe, 2015). As per Donaldson et al. (2000) and by Issel and Narasimha (2007), our study supports the argument that OM is appropriate for critical events that provides life and death service as it encourages a focus on an organisations ability to observe, interpret, and respond to cues in an appropriate manner. Significantly, our study extends the possible benefits of onward integration of OM (through mindful data selection) and collective critical reflection in action (heuristic data evolution) henceforth initiating systematic timely continuous corrective actions to effectively manage critical events (Jordan et al., 2009; Oeij et al., 2018; King & Badham, 2018). The constructs of mindfulness were embedded in enhancing operational awareness of the environment, where preoccupation with failure influenced the decision process providing rapid responses in the successful completion of operational actions, critical for keeping the environment safe.

In addition the paper delivers practical contributions. The study provides a rare insider view of a rapid decision-making process during an adverse critical event. With increased interest in academic literature on the topic of adverse critical events and crisis management (Hällgren et al., 2018; Williams et al., 2017) the research will help prepare managers for the many adverse challenges that are occurring. It offers guidance on the executive and operational attributes necessary for actors managing a critical situation. Finally, a limitation of the study lies in the research methodology of ‘inquiry from the inside’- where the researcher is personally and actively involved has the potential to introduce a lack of objectivity given the researcher is not an independent observer (Iacono et al. (2009). To address this limitation, we suggest future research in other international critical event studies to determine the role data can play in managing the critical event using traditional methods to overcome any potential bias. Future research could also investigate data selection methodologies to complement the concept of data evolution and to further understand how the data is constructed and utilised in the management of critical event.

## Figures and Tables

**Fig. 9:**
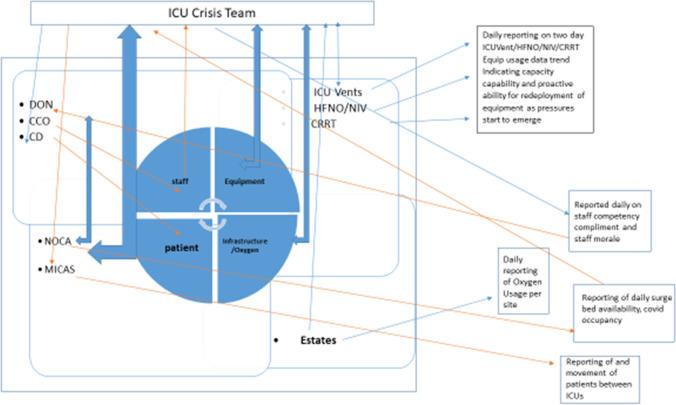
ICU crisis team data review process
